# Mechanical, Anisotropic, and Electronic Properties of XN (X = C, Si, Ge): Theoretical Investigations

**DOI:** 10.3390/ma10080912

**Published:** 2017-08-08

**Authors:** Zhenyang Ma, Xuhong Liu, Xinhai Yu, Chunlei Shi, Dayun Wang

**Affiliations:** Tianjin Key Laboratory for Civil Aircraft Airworthiness and Maintenance, Civil Aviation University of China, Tianjin 300300, China; liuxuhong16@163.com (X.L.); xhyucauc@126.com (X.Y.); clshi01@126.com (C.S.); dywang011@163.com (D.W.)

**Keywords:** C/Si/Ge-group-V compounds, electronic properties, mechanical properties, anisotropic properties

## Abstract

The structural, mechanical, elastic anisotropic, and electronic properties of *Pbca*-XN (X = C, Si, Ge) are investigated in this work using the Perdew–Burke–Ernzerhof (PBE) functional, Perdew–Burke–Ernzerhof for solids (PBEsol) functional, and Ceperly and Alder, parameterized by Perdew and Zunger (CA–PZ) functional in the framework of density functional theory. The achieved results for the lattice parameters and band gap of *Pbca*-CN with the PBE functional in this research are in good accordance with other theoretical results. The band structures of *Pbca*-XN (X = C, Si, Ge) show that *Pbca*-SiN and *Pbca*-GeN are both direct band gap semiconductor materials with a band gap of 3.39 eV and 2.22 eV, respectively. *Pbca*-XN (X = C, Si, Ge) exhibits varying degrees of mechanical anisotropic properties with respect to the Poisson’s ratio, bulk modulus, shear modulus, Young’s modulus, and universal anisotropic index. The (001) plane and (010) plane of *Pbca*-CN/SiN/GeN both exhibit greater elastic anisotropy in the bulk modulus and Young’s modulus than the (100) plane.

## 1. Introduction

In the last few decades, nitride-based ceramics such as silicon nitride (Si_3_N_4_) have attracted increasing attention from researchers in the ceramics, mechanical, and aerospace industries, as well as in fields such as solar cells, as they have a wide range of applications [1,2,3,4,5,6,7,8]. This is due to their significant chemical stability, good compression resistance, corrosion resistance, high hardness, good mechanical properties, and good optical performance characteristics. Other stoichiometries like Si_3_N_4_, SiN_2_, and Si_2_N_2_(NH) have also been proposed to exist [9,10,11,12,13,14]. Silicon and germanium-based compounds and alloys such as the Si/Ge-group-III and Si/Ge-group-V compounds have been widely investigated [15,16,17].

C*x*N*y* with different stoichiometries is often used as a potential superhard material [18,19,20,21]. Li et al. [18] have reported a novel body-centered tetragonal CN_2_ named bct-CN_2_, using the newly-developed particle swarm optimization algorithm for crystal structure prediction. They found that the hardness of *bct*-CN_2_ is 77.4 GPa, and it is an indirect wide gap semiconductor material with a band gap of 3.6 eV. Wang et al. [19] suggested a new carbon nitride phase consisting of *sp*^3^ hybridized bonds, with cubic symmetry and a *P*2_1_3 space group (i.e., *cg*-CN). Unlike most of the other superhard materials that are insulators or semiconductors, it is a metallic compound, and its Vickers hardness is 82.56 GPa. They found that *cg*-CN is the most favorable stable crystal structure, with carbon nitride with 1:1 stoichiometry. Using the particle swarm optimization technique, Wei et al. [20] proposed a cubic superhard phase of C_3_N (*c*-C_3_N) with a Vickers hardness of 65 GPa, which is more energetically favorable than the recently proposed *o*-C_3_N [21]. *o*-C_3_N was proposed by Hao et al. [21]. It has a *C*222_1_ phase, and its Vickers hardness is 76 GPa.

CN_2_, SiN_2_, and GeN_2_ were proposed by Manyali et al. [22] using first-principles calculations; they found that SiN_2_ and GeN_2_ both have mechanical stability, SiN_2_ and GeN_2_ are characterized by an indirect band gap, and the optical spectra of GeN_2_ is within the solar spectrum for CN_2_ and SiN_2_. The structural, elastic, electronic, and optical properties of Si_3_N_2_ [23] have been calculated using density functional theory. First, Si_3_N_2_ has both mechanical and dynamical stability at ambient pressure, and it is still stable at 10–20 GPa. The first-principles plane-wave pseudo-potential (PW-PP) method was applied to investigate the mechanical properties, thermal properties, and phase transition characters of Ge_3_N_4_ by Luo et al. [24]. The *β*→wII→*γ* phase transitions of Ge_3_N_4_ were also successfully predicted by them; at 300 K, the calculated *P*_t_ of the *β*→wII transition is 10.7 GPa, and the calculated *P*_t_ of the *β*→*γ* transition is 14.26 GPa at 1200 K. The bulk moduli of *β*-Ge_3_N_4_, wII-Ge_3_N_4_, and *γ*-Ge_3_N_4_ are 179 GPa, 187 GPa, and 220 GPa, respectively. Pseudocubic-Si_3_P_4_ and Ge_3_P_4_ [25] were proposed by first-principles calculations for investigating the electronic, mechanical, and optical properties of pseudocubic-Si_3_P_4_ and Ge_3_P_4_. The bulk modulus and shear modulus of pseudocubic-Si_3_P_4_ and Ge_3_P_4_ are 76 GPa and 58 GPa, and 60 GPa and 47 GPa, respectively. In addition, pseudocubic-Si_3_P_4_ and Ge_3_P_4_ are both indirect and narrow band gap semiconductor materials, with band gaps of 0.24 eV and 0.13 eV, respectively.

Recently, Wei et al. [26] investigated the stability and electronic and mechanical properties of *Pbca*-CN using first-principles calculations. The electronic properties and elastic anisotropy in bulk modulus, shear modulus, and Poisson’s ratio of *Pbca*-CN are not fully represented. We proposed *Pbca*-SiN and *Pbca-*GeN (space group: *Pbca*), which have a structure based on *Pbca-*CN, with silicon atoms or germanium atoms substituting carbon atoms. In this work, the stability as well as structural, mechanical, electronic, and elastic anisotropy properties of *Pbca-*XN (X = C, Si, Ge) were systematically investigated.

## 2. Materials and Methods

The theoretical calculations were carried out using first-principles density functional theory (DFT) [27,28]. The calculations were performed using the Cambridge Serial Total Energy Package (CASTEP) code [29]. The generalized gradient approximation (GGA) parameterized by Perdew–Burke–Ernzerhof (PBE) [30] functional, Perdew–Burke–Ernzerhof for solids (PBEsol) [31] functional, and the local density approximation (LDA) parameterized by Ceperly and Alder, parameterized by Perdew and Zunger (CA-PZ) [32,33] exchange-correlation functional were employed for the self-consistent total energy calculations and geometry optimization. The C/Si/Ge: 2*s*^2^2*p*^2^/3*s*^2^3*p*^2^/4*s*^2^4*p*^2^ and N: 2*s*^2^2*p*^3^ electrons were explicitly treated as valence electrons. The energy cutoff for the plane wave basis set was chosen to be 520/500/440 eV for CN/SiN/GeN in the *Pbca* phase. The conjugate gradient method was used for the relaxation of structural parameters. The *k*-point samplings with 2π × 0.025 Å^−1^ (7 × 9 × 10/5 × 7 × 8/5 × 7 × 8) in the Brillouin zone were performed using the Monkhorst–Pack scheme [34] for CN/SiN/GeN in *Pbca* phase. The structural parameters optimizations were determined using the Broyden–Fletcher–Goldfarb–Shenno (BFGS) algorithm [35], with the flowing thresholds for converged structures: energy change less than 5 × 10^−6^ eV per atom, residual force below 0.01 eV/Å, stress less than 0.02 GPa, and displacement of atoms during the geometry optimization less than 0.0005 Å. The phonon frequencies were calculated using linear response theory [36]. The electronic band structures of the CN/SiN/GeN in *Pbca* phase were calculated utilizing the Heyd–Scuseria–Ernzerhof (HSE06) [37,38] hybrid functional.

## 3. Discussion

### 3.1. Structural Properties

**Figure 1:**
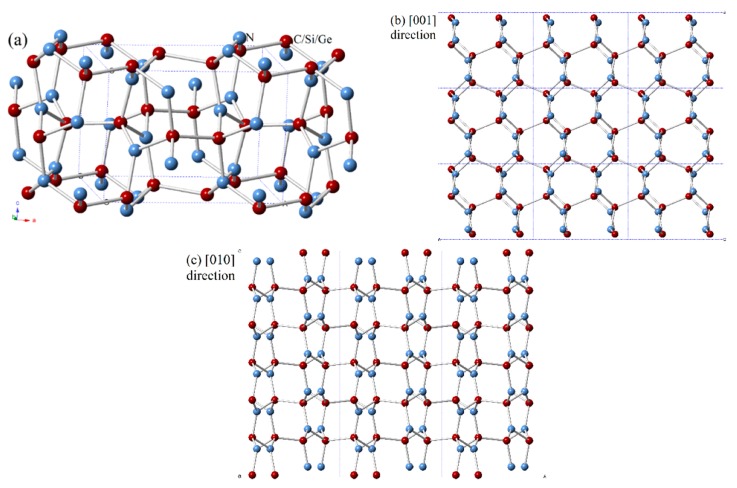
The crystal structures of (**a**) CN/SiN/GeN in the *Pbca* phase; and CN/SiN/GeN in the *Pbca* phase along the (**b**) (001) direction and (**c**) (010) direction. The blue and red spheres represent the N atoms and C/Si/Ge atoms.

**Figure 2:**
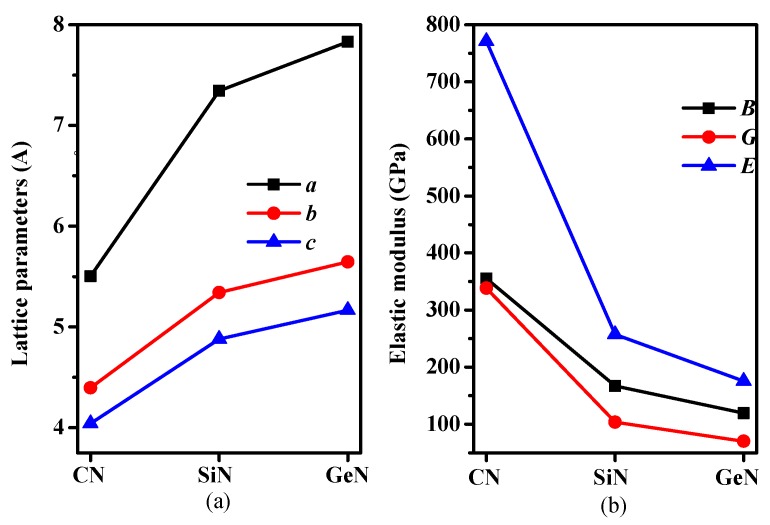
(**a**) Lattice parameters and (**b**) elastic moduli for *Pbca*-CN/SiN/GeN with PBE level.

In the newly-formed (*Pbca* phase) solid, all the nitrogen atoms have *sp*^2^ hybridizations and all the carbon/silicon/germanium atoms have *sp*^3^ hybridization with their nearest neighboring N and C/Si/Ge atoms. The crystal structures of CN/SiN/GeN in *Pbca* phase are shown in Figure 1a. The C/Si/Ge atoms and N atoms consist of zigzag six-membered rings and eight-membered rings. The C/Si/Ge and N atoms are located at Wyckoff 8*c* (0.1396, 0.0722, 0.0205)/(0.1507, 0.0794, 0.0291)/(0.1476, 0.0769, 0.0310) and 8*c* (0.8154, 0.8661, 0.6318)/(0.8038, 0.8690, 0.6241)/(0.8093, 0.8725, 0.6197) sites in *Pbca*-CN/SiN/GeN, respectively. The crystal structures of CN/SiN/GeN in the *Pbca* phase along the (001) direction and (010) direction are shown in Figure 1b,c, respectively. The eight-membered rings are normal to the (001) direction in the structure of *Pbca*-CN/SiN/GeN, and the six-membered rings are normal to the (010) direction. The optimal lattice parameters of *Pbca*-CN/SiN/GeN, together with the previous results [27,39] of *Pbca*-CN are listed in Table 1. The optimized lattice parameters are *a* = 5.504 Å, *b* = 4.395 Å, and *c* = 4.041Å, which are in excellent agreement with [27,39]. In addition, taking into account the van der Waals forces, we also calculated the lattice parameters of *Pbca*-CN/SiN/GeN and diamond, *c*-BN using the dispersion-corrected Perdew–Burke–Ernzerhof (PBE + D) [40]. For diamond and *c*-BN, the theoretical results obtained by the GGA-PBE level (diamond: 3.566 Å for PBE level, 3.526 Å for CA-PZ [41], experimental value 3.567 Å [42]; *c*-BN: 3.626 Å for PBE level, 3.569 Å for CA-PZ [43], experimental value 3.620 Å [44]) are closer to the experimental values; the obtained results of *c*-BN and diamond using PBE + D are not much different from those obtained by PBE functional compared to corresponding experimental values, so the results obtained by the GGA-PBE level are all used in our paper. The lattice parameters of *Pbca*-XN with X changing from C to Ge are illustrated in Figure 2a. It is clear that the lattice parameters of *Pbca*-XN increase with X changing from C to Ge. From CN to SiN, the lattice parameters increase 31.4%, 21.52%, and 20.7% for *a*, *b*, and *c* of SiN compared to CN, while the lattice parameters increase 8.25%, 5.69%, and 5.84% for *a*, *b*, and *c* of GeN compared to SiN, respectively. This is because the average bond length of Si-N (1.751 Å) is much greater than that of the C–N bond (1.452 Å), and the average bond length of Ge-N (1.871 Å) is slightly longer than that of the Si–N bond.

### 3.2. Mechanical Properties

**Figure 3:**
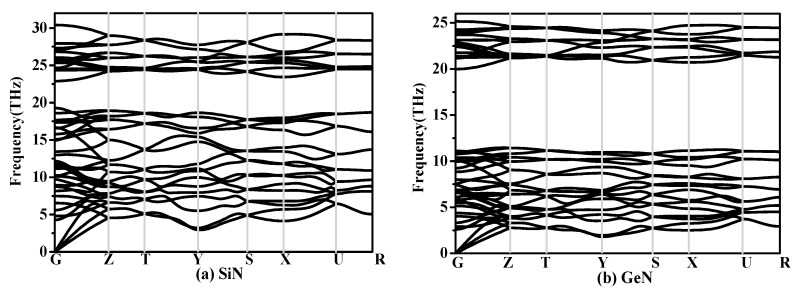
The phonon spectra of (**a**) *Pbca*-SiN and (**b**) *Pbca*-GeN with PBE level.

The calculated elastic constants and elastic moduli of CN/SiN/GeN in the *Pbca* phase are listed in Table 2. The calculated elastic constants and elastic modulus of *Pbca*-CN are excellent agreement with the previous report [26]. For an orthorhombic phase, the criteria of mechanical stability are [45]: *C*_ii_ > 0, *i* = 1–6; *C*_11_*C*_22_ − *C*${}_{12}^{2}$ > 0; *C*_11_*C*_22_*C*_33_ + 2*C*_12_*C*_13_*C*_23_ − *C*_11_*C*${}_{23}^{2}$ − *C*_22_*C*${}_{13}^{2}$ − *C*_33_*C*${}_{13}^{2}$ > 0, where the *C*_ij_ is elastic constant of the material. The mechanical stability of a phase can be confirmed by using the elastic constants. The SiN/GeN in the *Pbca* phase both satisfy the above mechanical stability criteria. The SiN/GeN in the *Pbca* phase show mechanical stability under ambient pressure. The phonon dispersion curve can show dynamic stability; the phonon dispersion curves of SiN/GeN in the *Pbca* phase are illustrated in Figure 3. There is no imaginary frequency in the Brillouin zone, which means SiN/GeN in the *Pbca* phase can be dynamically stable under ambient pressure. The elastic moduli of *Pbca*-XN with X changing from C to Ge are illustrated in Figure 2b. It is clear that the elastic moduli of *Pbca*-XN decrease with X changing from C to Ge. The elastic constants and elastic moduli of other Si*_x_*N*_y_* compounds [22,46,47] are also listed in Table 2. The bulk modulus *B* of *Pbca*-SiN is slightly smaller than that of SiN_2_, *o*-Si_3_N_4_, and *t*-Si_3_N_4_, while it is slightly larger than Si_3_N_2_ and *t*-Si_3_N_4_. The shear modulus *G* and Young’s modulus *E* of *Pbca*-SiN are similar to the bulk modulus of *Pbca*-SiN. For *Pbca*-GeN, its bulk modulus is as large as that of GeN_2_. However, its shear modulus and Young’s modulus are slightly smaller than that of GeN_2_.

Brittleness and ductility of materials are important properties in crystal physics and engineering sciences. Pugh [48] proposed the ratio of bulk to shear modulus (*B*/*G*) as an indication of ductile verses brittle characters. If *B*/*G* > 1.75, the material is characterized by a ductile manner; otherwise, the material has a brittle character. The Poisson’s ratio *v* is consistent with *B*/*G*, but refers to brittle compounds, usually with a small *v* (less than 0.26) [49]. The *B*/*G* ratio of *Pbca*-CN/SiN/GeN is 1.12 (1.11 [26]), 1.63, and 1.70; it is revealed that *Pbca*-CN/SiN/GeN are all brittle materials, and *Pbca*-CN has the most brittleness. For Poisson’s ratio *v*, we obtained the same conclusion.

The Debye temperature (*Θ*_D_) is a fundamental physical property, and correlates with many physical properties of solids (e.g., specific heat and the thermal coefficient) [50]. Debye temperature *Θ*_D_ can be estimated by elastic moduli. The Debye temperature can be estimated from the average sound velocity by the following equation based on elastic constant evaluations [51]: *Θ*_D_ = (*h*/*k_B_*) (3*nρN*_A_/4*πM*)^1/3^*v*_m_, where *h* is the Planck constant, *k*_B_ is Boltzmann’s constant, *n* is the number of atoms in the molecule, *N*_A_ is the Avogadro number, *M* is the molecular weight, and *ρ* is the density. The average sound velocity *v*_m_ can be calculated as follows: *v*_m_ = [(2/*v*${}_{t}^{3}$ + 1/*v*${}_{1}^{3}$)/3]^−1/3^, where *v*_l_ = [(*B* + 4*G*/3)/*ρ*]^1/2^, and *v*_t_ = (*G*/*ρ*)^1/2^, where *B* and *G* are bulk modulus and shear modulus, *v*_l_ is the longitudinal sound velocity, and *v*_t_ is the transverse sound velocity. In addition, we can obtain the sound velocity in the main directions of a material according to the elastic constants. For the (001) propagation direction in orthorhombic symmetry, polarization direction (001)*v*_l_ = (*C*_33_/*ρ*)^1/2^, (100)*v*_t1_ = (*C*_55_/*ρ*)^1/2^, and (010)*v*_t2_ = (*C*_44_/*ρ*)^1/2^. For the (010) propagation direction, polarization direction (010)*v*_l_ = (*C*_22_/*ρ*)^1/2^, (100)*v*_t1_ = (*C*_66_/*ρ*)^1/2^, and (001)*v*_t2_ = (*C*_44_/*ρ*)^1/2^. For the (100) propagation direction, polarization direction (100)*v*_l_ = (*C*_11_/*ρ*)^1/2^, (010)*v*_t1_ = (*C*_66_/*ρ*)^1/2^, and (100)*v*_t2_ = (*C*_55_/*ρ*)^1/2^ [49,52].

The calculated results of Debye temperature, longitudinal sound velocity, and transverse sound velocity of *Pbca*-XN (X = C, Si, Ge) are all listed in Table 3. The densities of *Pbca*-XN (X = C, Si, Ge) are also listed in Table 3. For *Pbca*-XN (X = C, Si, Ge), in the (001) propagation direction, the (001) polarization direction has the largest sound velocity. The longitudinal sound velocity in the (010) propagation direction aligns with the (001) polarization direction, and the longitudinal sound velocity in the (100) propagation direction aligns with the (010) polarization direction. The longitudinal sound velocity is generally larger than the transverse sound velocity, mainly because the elastic constants that determine the longitudinal sound velocity are greater than those of the transverse sound velocity. In addition, for the same the propagation direction and polarization direction, the sound velocity decreases with X changing from C to Ge. Furthermore, the Debye temperature of *Pbca*-XN (X = C, Si, Ge) decreases with X changing from C to Ge. For *Pbca*-SiN, the Debye temperature is 863 K; it is slightly smaller than that of *m*-Si_3_N_4_ (892 K), *o*-Si_3_N_4_ (1107 K), and *t*-Si_3_N_4_ (949 K) [53]. The longitudinal sound velocity and transverse sound velocity of *Pbca*-XN (X = C, Si, Ge) are different along different directions; this shows that the sound velocity of *Pbca*-XN (X = C, Si, Ge) is also anisotropic.

### 3.3. Electronic Properties

**Figure 4:**
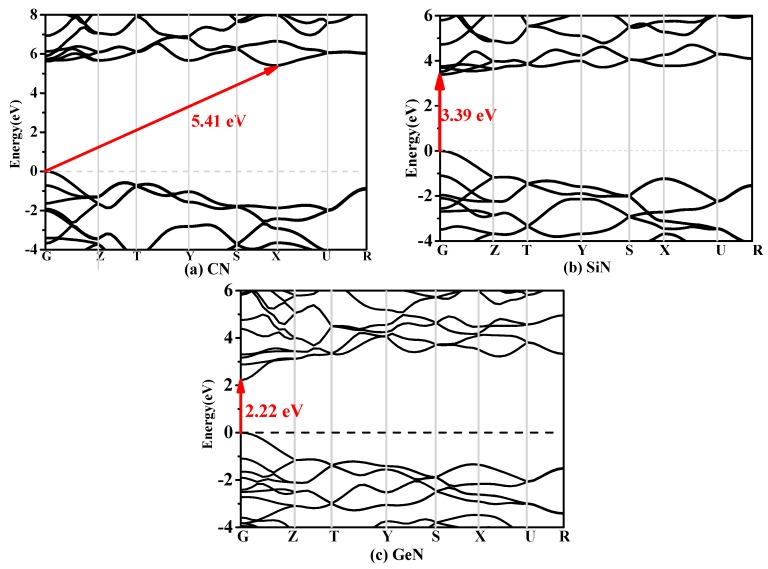
The band structures of (**a**) *Pbca*-CN; (**b**) *Pbca*-SiN; and (**c**) *Pbca*-CN GeN with the Heyd–Scuseria–Ernzerhof (HSE06) hybrid functional with PBE level.

In solid-state physics and semiconductor physics, the band structure of a solid or a material describes the energy that is forbidden or permitted by electrons. The band structure of a material determines a variety of properties—especially its electronic and optical properties. It is known that since the calculated band gap with DFT is usually underestimated by 30–50%, the band gap should be greater than the calculated results with the PBE functional. Hence, the band structures of *Pbca*-CN/SiN/GeN calculated utilizing the Heyd–Scuseria–Ernzerhof (HSE06) [37,38] hybrid functional are shown in Figure 4a–c, respectively. The band gap of *Pbca*-CN is 5.41 eV within the HSE06 hybrid functional and 3.96 eV within the PBE functional; the results of the PBE functional of *Pbca*-CN are in excellent agreement with previous report [26]. The valence band maximum is located at the G point in the Brillouin zone, whereas the conduction band minimum is located at the X point. That is to say, *Pbca*-CN is an indirect semiconductor with a band gap of 3.94 eV. In contrast, the valence band maximums of *Pbca*-SiN and *Pbca*-GeN are all located at the G point in the Brillouin zone; it is shown that *Pbca*-SiN and *Pbca*-GeN are both direct semiconductors with band gaps of 3.39 eV and 2.22 eV, respectively. In addition, the Fermi level decreases as the carbon atoms change into silicon atoms; the silicon atoms then change into germanium atoms, and the Fermi level also decreases. Similar to the lattice constants and the elastic moduli, the Fermi level changes rapidly when the carbon atom is replaced by silicon atoms. The Fermi levels of *Pbca*-CN/SiN/GeN are 11.02 eV, 2.26 eV, and 0.01 eV, respectively.

### 3.4. Elastic Anisotropy Properties

**Figure 5:**
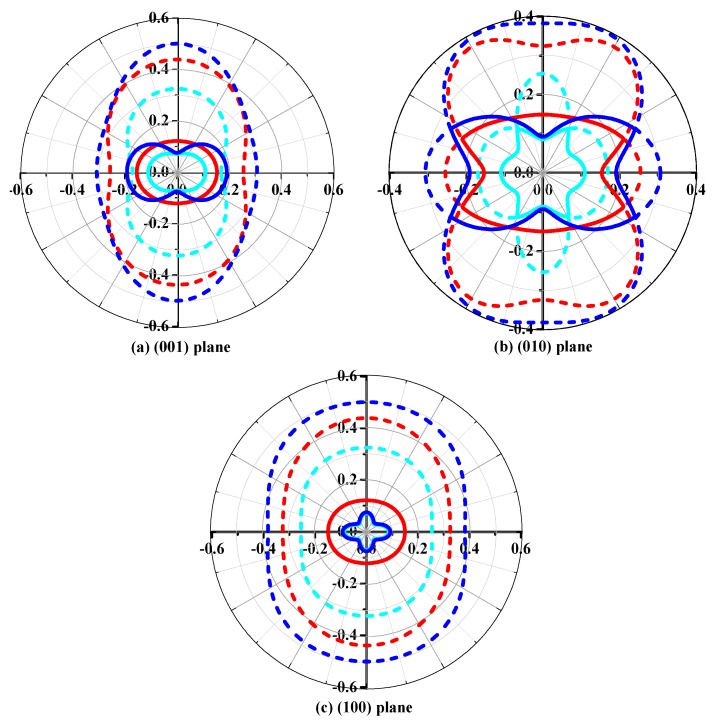
The two-dimensional representation of Poisson’s ratio in the (**a**) (001) plane; (**b**) (010) plane; and (**c**) (100) plane for *Pbca*-CN/SiN/GeN with PBE level. The cyan line, red line, and blue line represent the Poisson’s ratio *v* of *Pbca*-CN/SiN/GeN, respectively.

The elastic anisotropy properties are an important characteristic of materials. Along the different crystallographic directions, various elastic moduli exhibit different values. In this work, we mainly investigated the anisotropy of Poisson’s ratio *v*, shear modulus, bulk modulus, and Young’s modulus in different planes and different directions. The Poisson's ratio *v* and shear modulus *G* have two unit vectors (***a***, ***b***) and three angles [43,54], so they have a maximum value and a minimum value in the same direction, while the Young's modulus has only two unit vectors (***a***, ***b***) and a two-angle description [43,54], so it is in the same direction with only one value. The Poisson’s ratio *v* of *Pbca*-CN/SiN/GeN in the (001) plane (namely the *xy* or *ab* plane), the (010) plane (namely the *xz* or *ac* plane), and the (100) plane (namely the *yz* or *bc* plane) are displayed in Figure 5a–c, respectively. The dashed line and solid line represent the maximum value and minimum value of Poisson’s ratio in different directions in the (001) plane, (010) plane, and (100) plane; the cyan line, red line, and blue line represent the Poisson’s ratio *v* of *Pbca*-CN/SiN/GeN in (001) plane, (010) plane, and (100) plane in Figure 5, respectively. From Figure 5a–c, it is obvious that the Poisson’s ratio *v* of *Pbca*-CN/SiN/GeN exhibits a larger anisotropy. In the (001), (010), and (100) plane, along almost all directions, the *Pbca*-GeN exhibits the largest Poisson ratio. The positions of the maximum values are all located at *θ* = 1.57, *φ* = 4.73 (more details see [43,54]) for *Pbca*-CN/SiN/GeN; the angles *θ* and *φ* are measured in radians. The minimum values of *Pbca*-CN/SiN/GeN occupy the position *θ* = 2.33, *φ* = 1.87; *θ* = 1.46, *φ* = 1.06; and *θ* = 0.83, *φ* = 4.73, respectively.

**Figure 6:**
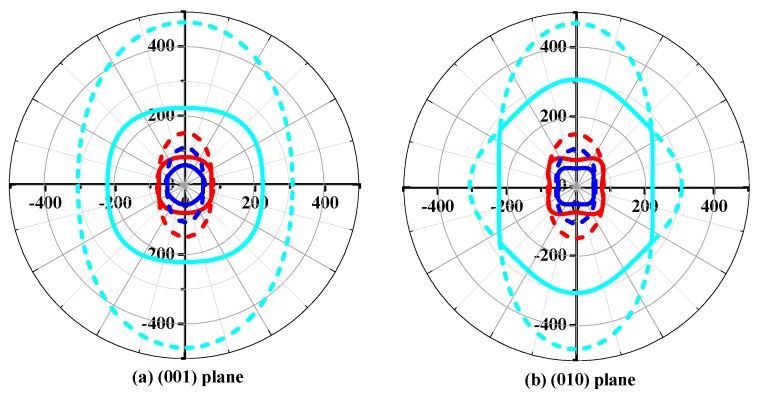

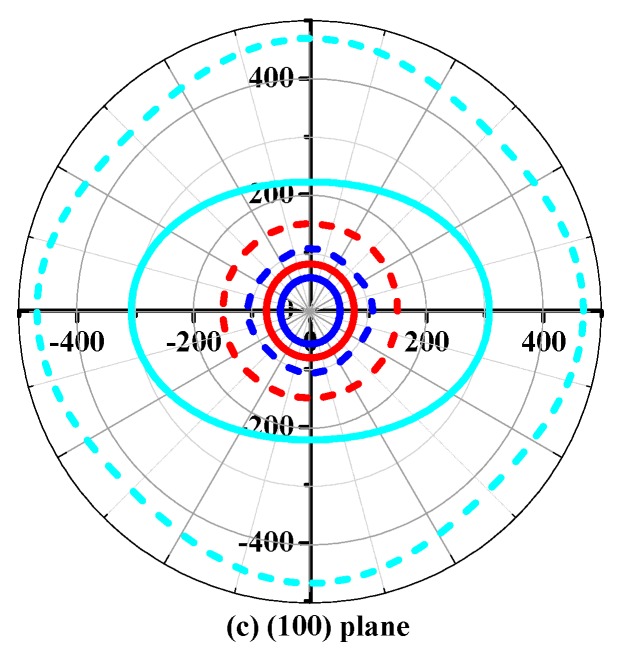
The two-dimensional representation of Young’s modulus in the (**a**) (001) plane; (**b**) (010) plane; and (**c**) (100) plane for *Pbca*-CN/SiN/GeN with PBE level. The cyan line, red line, and blue line represent the Poisson’s ratio *v* of *Pbca*-CN/SiN/GeN, respectively.

The shear moduli *G* of *Pbca*-CN/SiN/GeN in the (001), (010), and (100) planes are displayed in Figure 6a–c, respectively. The cyan line, red line, and blue line represent the Poisson’s ratio *v* of *Pbca*-CN/SiN/GeN in Figure 6, and the dashed line and solid line represent the maximum value and minimum value of the shear modulus, respectively. The maximum shear moduli *G* of *Pbca*-CN/SiN/GeN are 469 GPa, 150 GPa, and 107 GPa, and the minimum shear moduli of *Pbca*-CN/SiN/GeN are 379 GPa, 106 GPa and 74 GPa, respectively. From Figure 6a,c, with X change from C to Ge, the shape of the minimum value for shear modulus is increasingly rounded in the (001) plane and (100) plane, while in the (010) plane the shape of the minimum is closer to a square. The ratios *G*_max_/*G*_min_ of *Pbca*-CN/SiN/GeN are 1.24, 1.42, and 1.53; in other words, the elastic anisotropy in shear modulus becomes larger and larger with X changing from C to Ge.

**Figure 7:**
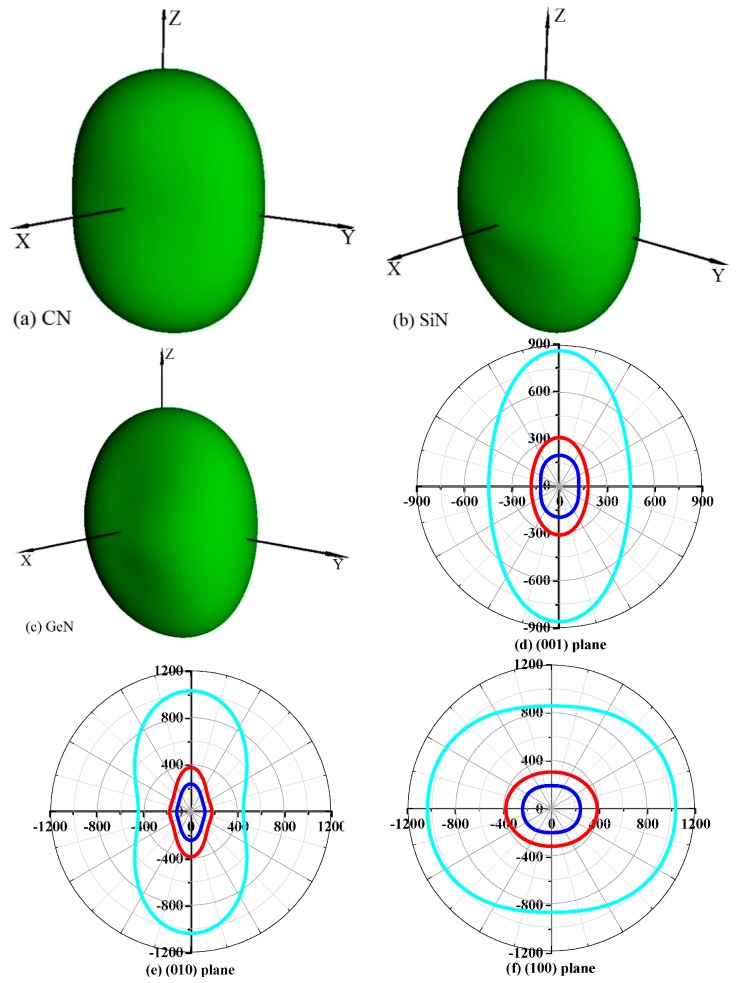
The surface constructions of Young’s modulus for (**a**) *Pbca*-CN; (**b**) *Pbca*-SiN; and (**c**) *Pbca*-GeN with PBE level. Two-dimensional representation of Young’s modulus in the (**d**) (001) plane; (**e**) (010) plane; and (**f**) (100) plane for *Pbca*-CN/SiN/GeN with PBE level. The cyan line, red line, and blue line represent the Poisson’s ratio *v* of *Pbca*-CN/SiN/GeN, respectively.

Young’s modulus is a measure of the stiffness of a solid material. It is a mechanical property of linear elastic solid materials. It defines the relationship between stress (force per unit area) and strain (proportional deformation) in a material. To study the elastic anisotropy in more detail, a variation of Young’s modulus with crystallographic direction is displayed in a three-dimensional manner. The directional dependence of Young’s modulus *E* for orthorhombic crystal is [55]: *E*^−1^ = *l*${}_{1}^{4}$*S*_11_ + *l*${}_{2}^{4}$*S*_22_ + *l*${}_{3}^{4}$*S*_33_ + 2*l*${}_{1}^{2}$*l*${}_{2}^{2}$*S*_12_ + 2*l*${}_{1}^{2}$*l*${}_{3}^{2}$*S*_13_ + 2*l*${}_{2}^{2}$*l*${}_{3}^{2}$*S*_23_ + *l*${}_{1}^{2}$*l*${}_{2}^{2}$*S*_66_ + *l*${}_{1}^{2}$*l*${}_{3}^{2}$*S*_55_ + *l*${}_{2}^{2}$*l*${}_{2}^{3}$*S*_44_, where *l*_1_, *l*_2_, and *l*_3_ are the direct cosines of the [*uvw*] direction, and *S*_ij_ refers to the elastic compliance constants. The three-dimensional surface representations of Young’s modulus *E* for *Pbca*-CN/SiN/GeN are illustrated in Figure 7a–c. For an isotropic system, the three-dimensional directional dependence exhibits a spherical shape. If there is a deviation of degrees from the spherical shape, it reflects the material exhibiting elastic anisotropy [56]. From Figure 7a–c, it is obvious that the shape of the three-dimensional directional dependence does not exhibit a spherical shape, and the shapes of the three-dimensional directional dependence for *Pbca*-CN/SiN/GeN all exhibit mechanical anisotropy in Young’s modulus.

**Figure 8:**
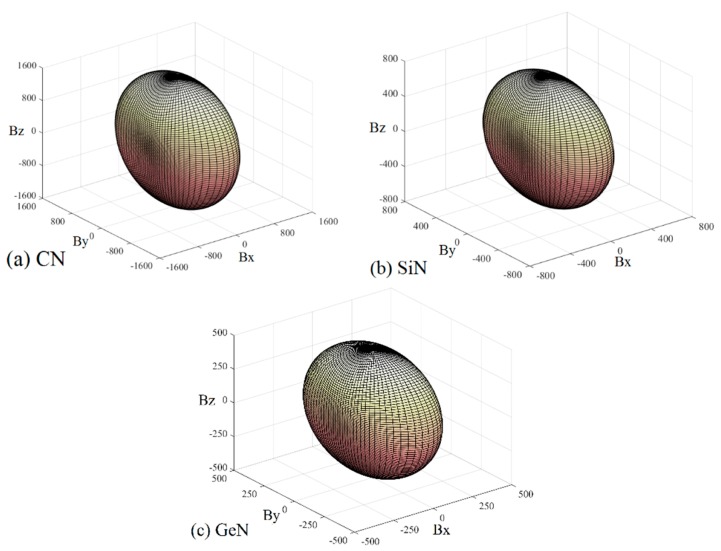
The surface constructions of bulk modulus in the (**a**) (001) plane; (**b**) (010) plane; and (**c**) (100) plane for *Pbca*-CN/SiN/GeN with PBE level.

To further understand the elastic anisotropy of the Young’s modulus along different directions, the dependence of the Young’s modulus on orientation is investigated when we take the tensile axis within a given plane. Let *α* be the angle of between (100) and (uv0) for the (001) plane; the Young’s modulus between (100) and (*uv*0) for the (001) plane can be expressed as: *E*^−1^ = *S*_11_cos^4^*α* + *S*_22_sin^4^*α +* 2*S*_12_sin^2^*α*cos^2^*α + S*_66_sin^2^*α*cos^2^*α*. Let *β* be the angle of between (001) and (*u0w*) for the (010) plane; the Young’s modulus between (001) and (*u0w*) for the (010) plane can be calculated as: *E*^−1^ = *S*_11_sin^4^*β* + *S*_33_cos^4^*β +* (2*S*_13_sin^2^2*β + S*_55_sin^2^2*β*)/4. Let *γ* be the angle of between (001) and (*0vw*) for the (001) plane, the Young’s modulus between (001) and (*0vw*) for the (001) plane can be estimated as: *E*^−1^ = *S*_22_sin^4^*γ* + *S*_33_cos^4^*γ +* (2*S*_23_sin^2^2*γ + S*_44_sin^2^2*γ*)/4. The two-dimensional representations of Young’s modulus in the (001) plane, (010) plane, and (100) plane for *Pbca*-CN/SiN/GeN are illustrated in Figure 7d–f, respectively. The cyan line, red line, and blue line represent the Poisson’s ratio *v* of *Pbca*-CN/SiN/GeN, respectively. From Figure 7d–f, the (001) plane and (010) plane of *Pbca*-CN/SiN/GeN exhibit a larger elastic anisotropy in Young’s modulus than the (100) plane. *Pbca*-CN has a maximum of *E*_max_ = 1034 GPa and a minimum of *E*_min_ = 447 GPa. The calculated results of elastic anisotropy in Young’s modulus for *Pbca*-CN are in excellent agreement with [26]. *Pbca*-CN/SiN/GeN has a maximum of *E*_max_ = 380/241 GPa and a minimum of *E*_min_ = 179/120 GPa. In order to quantify the elastic anisotropy, we introduce a ratio; that is, the ratio of the maximum and minimum Young’s modulus (ratio *E*_max_/*E*_min_). The greater the ratio *E*_max_/*E*_min_, the greater the maximum and minimum differences, and the greater the anisotropy of the material. Through the values of the ratio *E*_max_/*E*_min_ = 2.31, 2.12, and 2.01, it is shown that the elastic anisotropy in Young’s modulus for *Pbca*-XN (X = C, Si, Ge) decreases with X changing from C to Ge. In addition, the maximum values of *Pbca*-CN/SiN/GeN all occupy the position *θ* = 0, *φ* = 0; that is, the maximum values of *Pbca*-XN (X = C, Si, Ge) all occupy the *z* (*c*) axis, while the minimum values of *Pbca*-CN/SiN/GeN do not occupy the same position (*x* (*a*) axis). The minimum value of *Pbca*-SiN is located at *θ* = 1.32, *φ* = 0, but the minimum value of *Pbca*-CN/GeN occupies the position of *θ* = *π*/2, *φ* = 0. For the orthorhombic phase, the dependence of the bulk modulus *B* along the crystallographic direction is expressed by: *B*^−1^ = (*S*_11_ + *S*_12_ + *S*_13_)*l*_1_ + (*S*_12_ + *S*_22_ + *S*_23_)*l*_2_ + (*S*_13_ + *S*_23_ + *S*_33_)*l*_3_. The three-dimensional surface representations of bulk modulus *B* for *Pbca*-CN/SiN/GeN are illustrated in Figure 8a–c. The anisotropy of the bulk modulus of *Pbca*-XN (X = C, Si, Ge) is similar to that of Young’s modulus; the (001) plane and (010) plane of *Pbca*-CN/SiN/GeN exhibit a larger elastic anisotropy in bulk modulus than the (100) plane.

In addition, apart from the Poisson’s ratio, shear modulus, and Young’s modulus, there is another significant physical quantity which describes the elastic anisotropy of a material: the universal anisotropic index *A*^U^ [57], which is defined as *A*^U^ = 5*G*_V_/*G*_R_ + *B*_V_/*B*_R_ − 6, where *G* and *B* are the shear modulus and bulk modulus, and the subscripts V and R denote the Voigt and Reuss approximations, respectively. The calculated universal anisotropic indices of *Pbca*-XN (X = C, Si, Ge) are 0.717, 0.671, and 0.662, respectively. The elastic anisotropy in the universal anisotropic index *A*^U^ of *Pbca*-XN (X = C, Si, Ge) is similar to the bulk modulus, Young’s modulus, and shear modulus; it also decreases with X changing from C to Ge. Furthermore, for *Pbca*-CN, the universal anisotropic index is slightly smaller than that of *m*-C_3_N_4_ (0.798 [58]), while it is much higher than that of *t*-C_3_N_4_ (0.305 [58]). The universal anisotropic index of *Pbca*-SiN is slightly larger than that of *o*-Si_3_N_4_ (0.582 [49]), but it is smaller than that of *m*-Si_3_N_4_ (0.968) and *t*-Si_3_N_4_ (1.231) [49].

## 4. Conclusions

The structural, mechanical, electronic, and elastic anisotropy properties of CN, SiN, and GeN in orthorhombic phase were performed using DFT calculations in this work. SiN and GeN are mechanically and dynamically stable, fulfilling the Born stability criteria for an orthorhombic phase and phonon spectra, respectively. PBE function predicts lattice parameters that agree well with the previous report. From band gap calculations with the HSE06 function, SiN and GeN are direct band gap semiconductor materials with band gap of 3.39 eV and 2.22 eV, while CN has an indirect band gap with band gap of 5.41 eV. The elastic moduli of *Pbca*-XN (X = C, Si, Ge) such as Young’s moduli, bulk moduli, shear moduli, Poisson’s ratio, and sound velocities have also been reported in this work. The Debye temperature, longitudinal sound velocities, and transverse sound velocities are also estimated using the elastic constants. The elastic anisotropy calculations showed that *Pbca*-XN (X = C, Si, Ge) exhibited anisotropy in bulk modulus, shear modulus, Poisson’s ratio, Young’s modulus, and *A*^U^. Besides, the elastic anisotropy in bulk modulus, shear modulus, Poisson’s ratio, Young’s modulus, and *A*^U^ for *Pbca*-XN (X = C, Si, Ge) decreases with X changing from C to Ge.

## Figures and Tables

**Table 1:** The lattice parameters (in Å) of *Pbca*-CN/SiN/GeN using different functionals.

Materials	PBE			PBEsol			CA-PZ			PBE + D		
	*a*	*b*	*c*	*a*	*b*	*c*	*a*	*b*	*c*	*a*	*b*	*c*
CN	5.504	4.395	4.041	5.461	4.384	4.029	5.402	4.352	3.998	5.484	4.385	4.029
	5.514 ^1^	4.396	4.041									
	5.514 ^2^	4.396	4.041									
SiN	7.234	5.341	7.226	7.339	5.333	4.867	7.226	5.257	4.798	7.281	5.322	4.856
GeN	7.831	5.645	7.578	7.823	5.625	5.136	7.578	5.488	5.011	7.744	5.610	5.129
*c*-BN	3.626			3.612			3.569			3.600		
Diamond	3.566			3.558			3.526			3.566		

^1^ Ref [26]; ^2^ Ref [39]. CA-PZ: Ceperly and Alder, parameterized by Perdew and Zunger; PBE: Perdew–Burke–Ernzerhof; PBEsol: Perdew–Burke–Ernzerhof for solids.

**Table 2:** The calculated elastic constants *C*_ij_ (in GPa) and bulk moduli *B* (in GPa), shear moduli *G* (in GPa), Young’s moduli *E* (in GPa), and Poisson’s ratio *v* of *Pbca*-CN/SiN/GeN and other C*_x_*N*_y_*, Si*_x_*N*_y_*, and Ge*_x_*N*_y_* compounds with PBE level.

Materials	*C*_11_	*C*_12_	*C*_13_	*C*_22_	*C*_23_	*C*_33_	*C*_44_	*C*_55_	*C*_66_	*B*	*G*	*E*	*v*
CN	491	169	139	922	122	1080	469	307	222	356	319	771	0.139
CN ^1^	495	174	145	934	124	1112	465	313	243	363	326	754	0.154
SiN	221	107	88	367	90	422	150	75	81	170	104	257	0.243
SiN_2_^2^	836					1269	397		313	407	386	879	0.140
SiN_2_^3^	442	75	58	610	133	133	237	76	71	191	138	333	0.200
Si_3_N_2_^4^	261	97					68			152	73	190	0.290
*o*-Si_3_N_4_^5^	581	181	55	587	132	483	244	88	197	262	179	436	0.221
*t*-Si_3_N_4_^5^	277	152	145			312	178		207	194	126	311	0.233
*m*-Si_3_N_4_^5^	241	39	139	457	55	358	88	128	86	165	104	259	0.239
GeN	159	85	69	243	55	272	107	51	57	119	70	176	0.255
GeN_2_^3^	260	40	22	350	94	145	138	45	44	119	85	205	0.210
*o*-Ge_3_N_4_^6^										203	122	305	0.250
*t*-Ge_3_N_4_^6^										147	87	218	0.253
*m*-Ge_3_N_4_^6^										124	73	183	0.254

^1^ Ref [26]; ^2^ Ref [18]; ^3^ Ref [22]; ^4^ Ref [23]; ^5^ Ref [46]; ^6^ Ref [47].

**Table 3:** The density (in g/cm^3^), sound velocities (in m/s), average sound velocity (in m/s), and the Debye temperature (in K) for *Pbca*-CN/SiN/GeN with PBE level.

Materials		CN	SiN	GeN
*ρ*		3.536	2.922	5.039
(100)	(100)*v_l_*	11,784	8697	5617
	(010)*v*_*t*l_	7927	5265	3363
	(001)*v*_*t*2_	9318	5066	3181
(010)	(010)*v_l_*	16,148	11,207	6944
	(100)*v*_*t*1_	7927	5265	3363
	(001)*v*_*t*2_	11,517	7165	4608
(001)	(001)*v_l_*	17,477	12,018	7347
	(100)*v*_*t*1_	9318	5066	3181
	(010)*v*_*t*2_	11,517	7165	4608
*v*_l_		14,865	10,278	6491
*v*_t_		9498	5966	3727
*v*_m_		10,437	6620	4140
*Θ*_D_		1702	863	508
